# GABA_A_ receptor occupancy by subtype selective GABA_Aα2,3_ modulators: PET studies in humans

**DOI:** 10.1007/s00213-016-4506-4

**Published:** 2016-12-24

**Authors:** Aurelija Jucaite, Zsolt Cselényi, Jaakko Lappalainen, Dennis J. McCarthy, Chi-Ming Lee, Svante Nyberg, Katarina Varnäs, Per Stenkrona, Christer Halldin, Alan Cross, Lars Farde

**Affiliations:** 1 Department of Clinical Neuroscience, AstraZeneca PET Center, Karolinska Institutet, R5:02, SE-17176 Stockholm, Sweden; 2 Department of Clinical Neuroscience, PET Centre, Karolinska Institutet, Stockholm, Sweden; 3 AstraZeneca Neuroscience Innovative Medicines, Cambridge, MA USA; 4 Marinus Pharmaceuticals, Radnor, PA USA; 5 AstraZeneca R&D, Södertälje, Sweden; 6 Independent Consultant, Newark, DE USA; 7 Ever East Consultants Limited, Hong Kong, People’s Republic of China; 8 Department of Psychiatry, Karolinska University Hospital (Huddinge), Stockholm, Sweden

**Keywords:** GABA_A_ receptors, PET, Anxiety, Agonist, AZD6280, AZD7325, [^11^C]flumazenil

## Abstract

**Rationale:**

Sedation, dependence, and abuse liability limit the use of non-selective γ-aminobutyric acid (GABA_A_) receptor positive modulators for the treatment of anxiety. AZD7325 and AZD6280 are novel, subtype-selective GABA_Aα2,3_ receptor positive modulators with limited sedative effects.

**Objectives:**

The current study aimed to confirm target engagement at GABA_A_ receptors by AZD7325 and AZD6280 in humans and to determine the relationship between exposure, GABA_A_ receptor occupancy, and tolerability.

**Method:**

Two PET studies, using high-resolution research tomography (HRRT) and the radioligand [^11^C]flumazenil, were performed in 12 subjects at baseline and after administration of single oral doses of AZD7325 (0.2 to 30 mg) and AZD6280 (5 to 40 mg). PET images were analyzed using a simplified reference tissue model, and regional binding potentials (BP_ND_) were obtained. The relationship between plasma concentration of AZD7325 or AZD6280 and GABA_A_ receptor occupancy was described by hyperbolic function, and *K*_i,plasma_ (plasma concentration required for 50% receptor occupancy) was estimated. Assessments of safety and tolerability included recording of adverse events, vital signs, electrocardiogram, and laboratory tests.

**Results:**

The [^11^C]flumazenil binding was reduced in a dose-dependent, saturable manner by both agents. Maximum receptor occupancy could be reached for both compounds without causing sedation or cognitive impairment. The *K*_i,plasma_ estimates for AZD7325 and AZD6280 were 15 and 440 nmol/l, respectively.

**Conclusion:**

High GABA_A_ receptor occupancy by AZD7325 and AZD6280 could be reached without clear sedative effects.

**Electronic supplementary material:**

The online version of this article (doi:10.1007/s00213-016-4506-4) contains supplementary material, which is available to authorized users.

## Background

Benzodiazepines (BZs), discovered by serendipity in the 1950s (Sternbach 1979), are well-established medicines for the treatment of anxiety (Nemeroff 2003). Its anxiolytic effects and related agents are mediated by allosteric enhancement of the γ-aminobutyric acid, GABA_A_ receptor complex thus potentiating inhibitory GABA-mediated neurotransmission (Farb and Ratner 2014, review). This mechanism of action of BZs has led to hypotheses on a pivotal role for GABA neurotransmission in the pathophysiology of anxiety (Nutt et al. 1990; Tiihonen et al. 1997; Malizia et al. 1998).

Although benzodiazepines are rapid onset highly efficacious drugs, significant adverse effects, such as sedation, amnesia, ataxia, abuse, dependence, and withdrawal, limit their clinical usefulness in long-term treatment of anxiety disorders. Accordingly, they are now reserved for second-line treatment (Baldwin et al. 2014). Building on an understanding of the pharmacological profile of the first-generation benzodiazepines, effective, safer, and tolerable GABA_A_ receptor modulators are now being sought (Skolnick 2012).

There is potentially a great diversity in the GABA_A_ receptor system at a detailed molecular level, as these ionotropic receptors are pentamers made up from 19 known subunits (α1–6, β1–3, γ1–3, δ, ε, θ, π, and ρ1–3). In the human brain, the majority of combinations consist of two α, two β, and one γ subunit (Olsen and Sieghart 2009). Benzodiazepine-like modulators of GABA_A_ receptors bind to a site, located on the α/γ subunit interface (Trincavelli et al. 2012, review), and pharmacological responses to BZ site activators vary with specific subunit composition. Studies with transgenic mice with introduced point mutations in murine α1 subunit gene have led to the association of anxiolytic-like activity with the GABA_A_ receptor α2 and/or α3 subunits, sedation with α1 subunit, and some aspects of cognition to the GABA_A_ receptor α5 subunit (Rudolph et al. 1999). Thus, it has been hypothesized that benzodiazepine site modulators which selectively potentiate activity of GABA_A_ receptors containing α2/α3 receptor subunits would produce a greater separation between therapeutic and side effects than non-selective compounds. In parallel, it has been hypothesized that side effects of BZs may be related to the intrinsic activity of modulators (Haefely et al. 1990; Puia et al. 1992); thus, partial agonism may also allow for differentiation of anxiolytic effects from unfavorable CNS effects such as sedation.

Based on these concepts, a series of subunit selective GABA_Aα2,3_ partial receptor modulators, including AZD7325 and AZD6280, were developed by AstraZeneca (Suppl. Fig. 1, Alhambra et al. 2011). Both compounds exert their function selectively via GABA_A_ receptor subunits as characterized by high in vitro affinity to the α1, α2, and α3 subunits and low affinity to the α5 subunit (Suppl. Table 1) and are partial BZ site modulators with efficacy selective for α2β3γ2 or α3β3γ2 subunits, i.e., AZD6280 produces 32–34% and AZD7325 produces 15% of maximal diazepam response, respectively (Chen et al. 2014, 2015, Suppl. Table 1). In preclinical models, these compounds have potent anxiolytic-like effects without sedation and induce a distinct pharmacoEEG signature (Alhambra et al. 2011; Christian et al. 2015). Examination of pharmacodynamic effects in phase I clinical trials has shown a novel pattern of effects on EEG (reduction in delta and theta bands) by AZD7325 and AZD6280, as well as effects on saccadic peak velocity, a suggested marker of anxiolysis (Chen et al. 2014, 2015).

The binding of BZs at the GABA_A_ receptor benzodiazepine binding site in humans has been examined extensively by PET using the radioligand [^11^C]flumazenil (Persson et al. 1985; Pike et al. 1993; Abadie et al. 1996). Flumazenil is an antagonist with high affinity for the GABA_A_ α1, α2, α3, and α5 subunits (*K*_i_ ∼1 nmol/l), whereas the affinity is lower for the α4 and α6 subunits (*K*_i_ ∼150 nmol/l) (Sieghart 1995). [^11^C]flumazenil continues to be used as a tool in research on the pathophysiology of neuropsychiatric disorders as well as for examination of GABA_A_ receptor occupancy by classical and novel receptor modulators.

We aim to confirm target GABA_A_ receptor engagement in humans by the modulators AZD7325 and AZD6280 and examine the relationship to sedation and previously published pharmacodynamics effects. Twelve subjects were examined using high-resolution research tomography (HRRT) and the radioligand [^11^C]flumazenil. For dose finding purposes, we examined the relationships between dose, plasma concentration, and receptor occupancy for both compounds.

## Method

### Study design and subjects

Two separate open-label phase I PET studies were conducted in 2008–2009. In the first study (study 1), we examined receptor occupancy at GABA_A_ receptors after administration of AZD7325 and in the second study (study 2) after administration of AZD6280.

The studies were approved by the Medical Products Agency of Sweden, the Regional Ethical Review Board in Stockholm, and the Radiation Safety Committee at the Karolinska University Hospital (KUH), Stockholm, Sweden. The studies were performed in accordance with the Declaration of Helsinki and International Conference on Harmonization/Good Clinical Practice Guidelines. Written informed consent was obtained from all subjects prior to the initiation of the study.

Subjects were enrolled and remained at the AstraZeneca Clinical Pharmacology Unit, KUH, Huddinge, for the duration of the study. Magnetic resonance imaging (MRI) and PET examinations were performed at KUH, Solna. Four men, 23–34 years of age, underwent repeated PET examinations with [^11^C]flumazenil in study 1, and eight men 21–32 years of age in study 2. The subjects were healthy according to medical history, physical examination, blood and urine analyses, and brain MRI. None of the subjects discontinued the study.

**Fig. 1:**
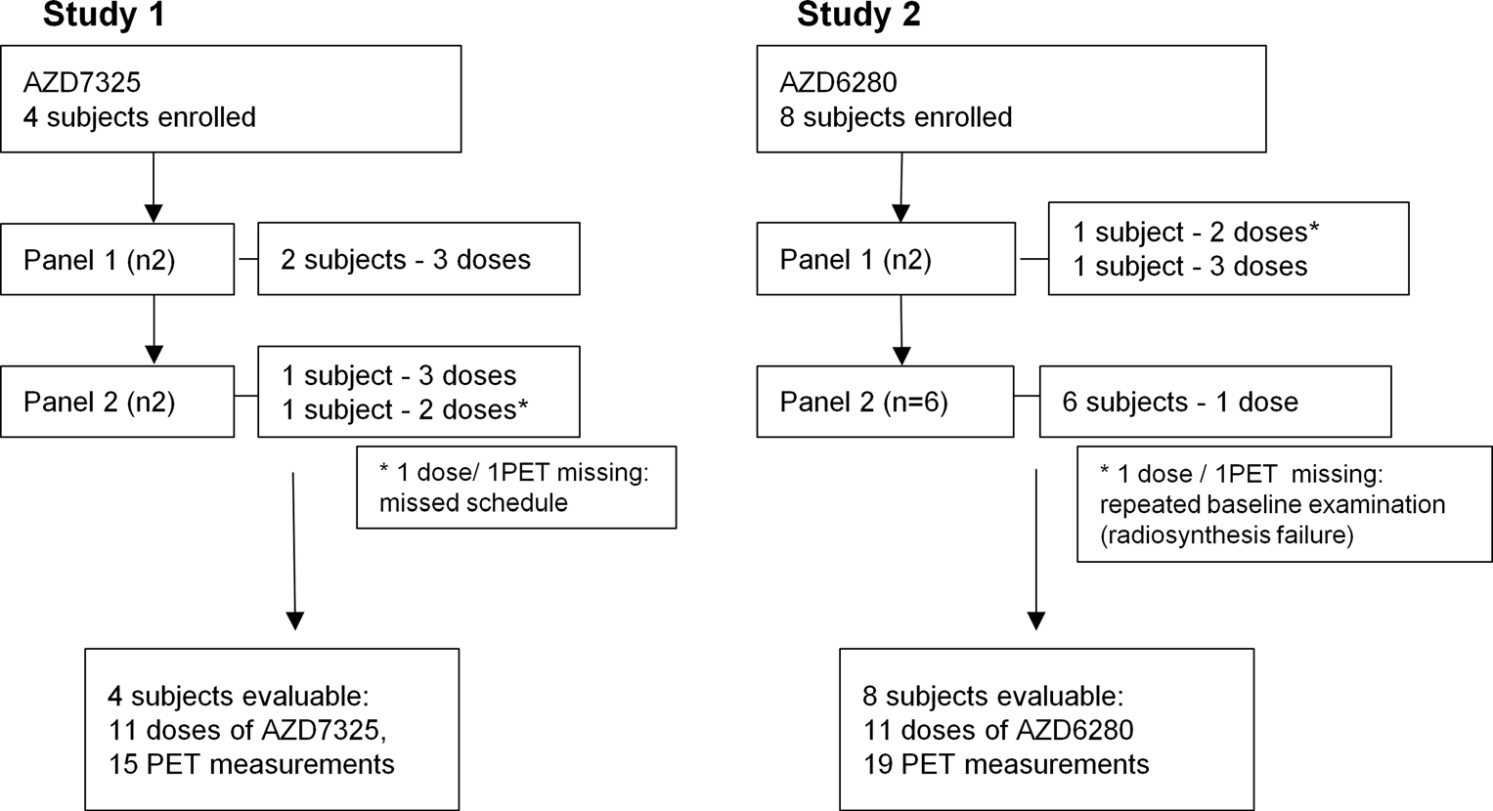
Flowchart of the two PET studies with GABA_A_ agonists AZD7325 and AZD6280

The initial study design included two sequential panels, with two subjects in each. The first panel was intended to obtain initial receptor occupancy values, to be used for dose selection in the second panel. Each of the subjects was planned to participate in four PET examinations with [^11^C]flumazenil, including one baseline assessment and subsequent weekly examinations at the approximate time of maximum drug plasma concentration, *T*_max_ (1 h), after administration of different single oral doses of AZD6280 or AZD7325. The studies followed an adaptive design, i.e., doses and PET measurements were adjusted depending on the results of preceding measurements. For AZD7325 (study 1), the second panel was run in line with the first panel, altogether providing occupancy values covering the saturation curve. For AZD6280 (study 2), due to the maximum tolerated dose of 40 mg of AZD6280 reached in the parallel multiple ascending dose study, the study design was re-arranged. To optimize quantification of the relationship between AZ6280 exposure and receptor occupancy, the number of subjects with the highest 40 mg dose was increased (Fig. 1).

### Study drugs, pharmacokinetic, and safety-tolerability measurements

Single doses of AZD7325 (doses of 0.2, 1, 2, 5, 20, and 30 mg) and AZD6280 (doses of 5, 12, 20, 30, and 40 mg) were administered as immediate release capsules (manufactured at AstraZeneca, Sweden) 60 min before PET measurements. The AZD7325 dose of 0.2 mg was prepared as an oral solution (0.2 mg) (powder manufactured at AstraZeneca, solution prepared at the Pharmacy of the KUH, Stockholm, Sweden).

Venous blood samples for determination of drug plasma concentrations were collected at approximately 15 min before [^11^C]flumazenil injection and at 15 and 30 min and 3, 4, 6, 8, 10, 12, 16, 24, 36, and 48 h after drug administration, including three samples during PET measurement, at 1, 1.30, and 2 h after drug administration. The plasma concentrations of AZD7325 and AZD6280 were determined by solid-phase extraction using Waters HLB μElution plate followed by LC-MS/MS (AstraZeneca Department of Development Drug Metabolism, Pharmacokinetics, and Bioanalysis in Wilmington, DE, USA).

Calculations of the pharmacokinetic parameters included the maximum plasma concentration (*C*_max_), the time to the maximum plasma concentration (*T*_max_), and the average drug plasma concentration during the PET measurement (*C*_av,PET_) (division of the partial area corresponding to the start and end times of PET assessment, AUC_PET_, by the duration of the PET measurement). The pharmacokinetic calculations were performed using non-compartmental analysis using WinNonlin™, Pharsight Corporation.

Safety and tolerability assessments included records of adverse events, vital signs (blood pressure, heart rate), Allen’s test, ECG, clinical chemistry, hematology assessments, and urinalysis.

### Imaging procedures

#### MRI measurements

Prior to PET measurements, 3D brain MRI examinations were acquired on a 1.5-T General Electric Signa system (GE, Milwaukee, WI, USA) at the MRI Center of KUH, Solna. Two examinations were made in one session. The T2-weighted images were acquired for clinical evaluation, and the T1-weighted images were used for delineation of anatomical brain regions of interests (ROIs). The T1 sequence was a 3D SPGR protocol in the axial plane with the following parameters: TR 23 ms, TE 4 ms, matrix 256 × 192 × 156, and voxel size 1.02 × 1.02 × 1.0 mm.

#### PET measurements

[^11^C]flumazenil was produced at the Karolinska Institutet PET Center by methylation of the corresponding desmethyl precursor analog using [^11^C]methyl triflate (Någren and Halldin 1998).

The head of the subject was fixed using individual plaster helmet. A sterile physiological phosphate buffer (pH 7.4) solution containing [^11^C]flumazenil was injected intravenously as a bolus during 2 s, immediately followed by flush with 10 ml saline. The injected radioactivity for [^11^C]flumazenil was in the range of 330–350 MBq, (340 (9) MBq, mean, SD) in the study 1 and in the range of 317–363 MBq (335 (16) MBq) in the study 2. The specific radioactivity of the radioligand at time of injection ranged from 310 to 1465 GBq/μmol (730 (414) GBq/μmol, mean, SD) and from 464 to 1879 GBq/μmol (977 (424) GBq/μmol, mean, SD), for study 1 and study 2, respectively.

PET examinations were performed over 63 min (HRRT; Siemens/CTI). List mode data were reconstructed using the ordinary Poisson 3D ordered subset estimation maximization algorithm, with 10 iterations and 16 subsets including modeling of the point spread function (Varrone et al. 2009) into a 4D PET image containing 33 consecutive time frames (9 × 10 s, 2 × 15 s, 3 × 20 s, 4 × 30 s, 4 × 60 s, 4 × 180 s, 7 × 360 s) with a 3D array of 256 × 256 × 207 voxels having a size of 1.22 × 1.22 × 1.22 mm. Attenuation correction was acquired with a 6-min transmission measurement using a single 137Cs source.

##### PET image analysis

The high-resolution T1-weighted MR images were re-oriented, re-sampled, and cropped to generate 220 × 220 × 170 matrix with 1-mm^2^ voxels. The T1-weighted MR images were co-registered to the PET images and re-sliced to a resolution of 2 × 2 × 2 mm, using SPM5 software (Wellcome Department of Cognitive Neurology, UK).

The anatomical brain regions were delineated manually on the MR images using in-house image analysis software. The regions included cortical and striatal subregions, thalamus, cerebellum, limbic regions, pons, and whole brain. The following four regions were selected in the final analysis: The occipital cortex and cerebellum were chosen because of the highest GABAA receptor binding by [^11^C]flumazenil and good imaging statistics and preferential distribution of α1/α2 receptor subtypes; the amygdala was chosen because of its role in the brain circuits involved in anxiety and putamen for preferential α1/α2/α3 versus α5 receptor subtypes (Lingford-Hughes et al. 2002; Fatemi et al. 2013; Waldvogel and Faull 2015). For the pons, the ROI was drawn on horizontal projection on the six central slices. The ROIs were displayed on the corresponding PET images, and the average concentration of radioactivity for the whole volume of anatomical structure was obtained by pooling the data from a series of sections. The concentration of radioactivity in each ROI, calculated for each sequential time frame and corrected for ^11^C decay, was plotted versus time (TACs).

An index of neuronal GABA_A_ receptor density or binding potential (BP_ND_) was calculated using the simplified reference tissue model (Lammertsma and Hume 1996) and pons as a reference region. The calculated receptor occupancy (%) was then correlated to drug exposure according to the following equation:

1 $$ \mathrm{Occupancy}=\left({Occ}_{\max}\times {C}_{av,\mathrm{PET}}\right)/\left({K}_{i,\mathrm{plasma}}+{C}_{av,\mathrm{PET}}\right) $$

where Occ_max_ is the maximal occupancy induced by drug, *C*_av,PET_ is the total drug plasma concentration, and *K*_i,plasma_ is the inhibition constant corresponding to the drug plasma concentration required for half-maximum receptor occupancy. Non-linear least squares curve fitting of the relationship between drug plasma concentration and receptor occupancy was performed using Matlab R2007b (http://de.mathworks.com). Regional data were fitted simultaneously to obtain a single estimate of *K*_i,plasma_ and regionally different estimates of Occ_max_. Best fit estimate and confidence interval (95% CI) were obtained on the logarithmic scale.

## Results

### Clinical observations

In the present study, there were no serious adverse events (AEs) related to either of the test drugs. Among subjects receiving AZD7325, CNS-related AEs such as dizziness, feeling hot, anhedonia, and hypoesthesia were reported by three subjects receiving the dose of 20 and 30 mg and in one subject both at 20 and at 5 mg (RO > 70%, Table 1). Among subjects receiving AZD6280, the most common AEs were dizziness (four of eight subjects) and feeling drunk (two out of eight subjects). The AEs were reported at doses of 20 mg and above (RO > 60%, Table 2), but not at 5 and 12 mg dose. No overt sedative-like effects were reported or observed. No consistent changes were observed in vital signs, ECG, clinical chemistry, hematology, or urinalysis variables. All AEs were of mild-to-moderate intensity and resolved without additional interventions.

**Table 1:** Pharmacokinetic parameters for AZD7325 and GABA_A_ receptor occupancy in four subjects

Subject	Dose (mg)	*C*_max_ (nmol/l)	*T*_max_ (h)	*C*_av,PET_ (nmol/l)	BP_ND_ (baseline)				Occupancy (%)			
					OC	CER	PUT	AMG	OC	CER	PUT	AMG
1	0.2	2.29	0.5	1.74	5.0	2.9	2.0	3.7	−8	−5	6	−12
2	0.2	4.12	0.5	2.93	5.9	3.4	2.2	4.2	0.5	3	−7	1
3	1	5.19	1.8	4.97	6.0	3.0	2.5	4.5	25	43	35	19
4	1	12.84	1.0	10.36	5.2	2.9	2.9	4.5	11	25	−3	−14
3	2	5.19	1.3	4.60	6.0	3.0	2.5	4.5	24	45	28	0.2
4	2	21.36	1.1	15.60	5.2	2.9	2.9	4.5	42	60	26	17
1	5	50.23	1.0	46.28	5.0	2.9	2.0	3.7	77	85	40	16
2	5	44.02	2.2	39.22	5.9	3.4	2.2	4.2	67	82	37	26
1	20	213.33	3.0	57.28	5.0	2.9	2.0	3.7	75	77	41	9
2	20	301.94	3.0	281.62	5.9	3.4	2.2	4.2	85	95	46	32
4	30	307.58	2.2	265.25	6.0	3.0	2.3	4.5	82	89	52	37

Receptor occupancy was measured at *T*_max_ of AZD7325, 1 h after drug administration

*C*_max_ maximum plasma concentration, *T*_max_ time for maximum plasma concentration, *C*_av,PET_ average plasma concentration at time of PET measurement, *BP*_*ND*_ binding potential, *OC* occipital cortex, *CER* cerebellum, *PUT* putamen, *AMG* amygdala

**Table 2:** Pharmacokinetic parameters for AZD6280 and GABA_A_ receptor occupancy in eight subjects

Subject	Dose (mg)	*C*_max_ (nmol/l)	*T*_max_ (h)	*C*_av,PET_ (nmol/l)	BP_ND_ (baseline)				Occupancy (%)			
					OC	CER	PUT	AMG	OC	CER	PUT	AMG
2	5	179.03	1.0	144.37	4.3	2.1	1.7	3.1	11	22	6	2
7	5	77.23	1.8	52.94	4.7	3.2	1.8	3.0	7	10	−5	−13
5	12	248.90	0.9	204.68	4.5	2.6	1.9	3.5	17	25	0	−8
8	20	676.82	1.6	592.22	5.3	2.6	2.1	4.2	60	65	32	20
1	30	728.67	0.5	513.07	5.2	3.0	2.0	4.1	39	50	16	9
2	30	731.40	1.6	676.82	4.3	2.1	1.7	3.1	43	59	14	5
1	40	676.82	1.0	570.39	5.2	3.0	2.0	4.1	50	69	26	18
2	40	1247.21	1.0	979.75	4.3	2.1	1.7	3.1	52	61	17	19
3	40	1495.56	0.5	1189.90	5.8	3.1	2.1	4.2	62	69	34	26
4	40	439.39	1.6	390.26	5.9	3.1	2.1	4.0	53	62	33	17
6	40	1514.66	0.6	750.51	3.8	2.6	1.6	3.0	36	60	10	−16

Receptor occupancy was measured at *T*_max_ of AZD7325, 1 h after drug administration

*C*_max_ maximum plasma concentration, *T*_max_ time for maximum plasma concentration, *C*_av,PET_ average plasma concentration at time of PET measurement, *BP*_*ND*_ binding potential, *OC* occipital cortex, *CER* cerebellum, *PUT* putamen, *AMG* amygdala

### Pharmacokinetic parameters

The range for peak exposures of AZD7325, *C*max, was from 0.81 to 109 nmol/l, and peak plasma concentrations were reached at 0.47 to 3 h. For AZD6280, *C*_max_ was in the range from 28.3 to 555 nmol/l. Peak plasma concentrations occurred in the time window from 0.5 to 1.77 h (Tables 1 and 2).

### GABA_A_ receptor occupancy

**Fig. 2:**
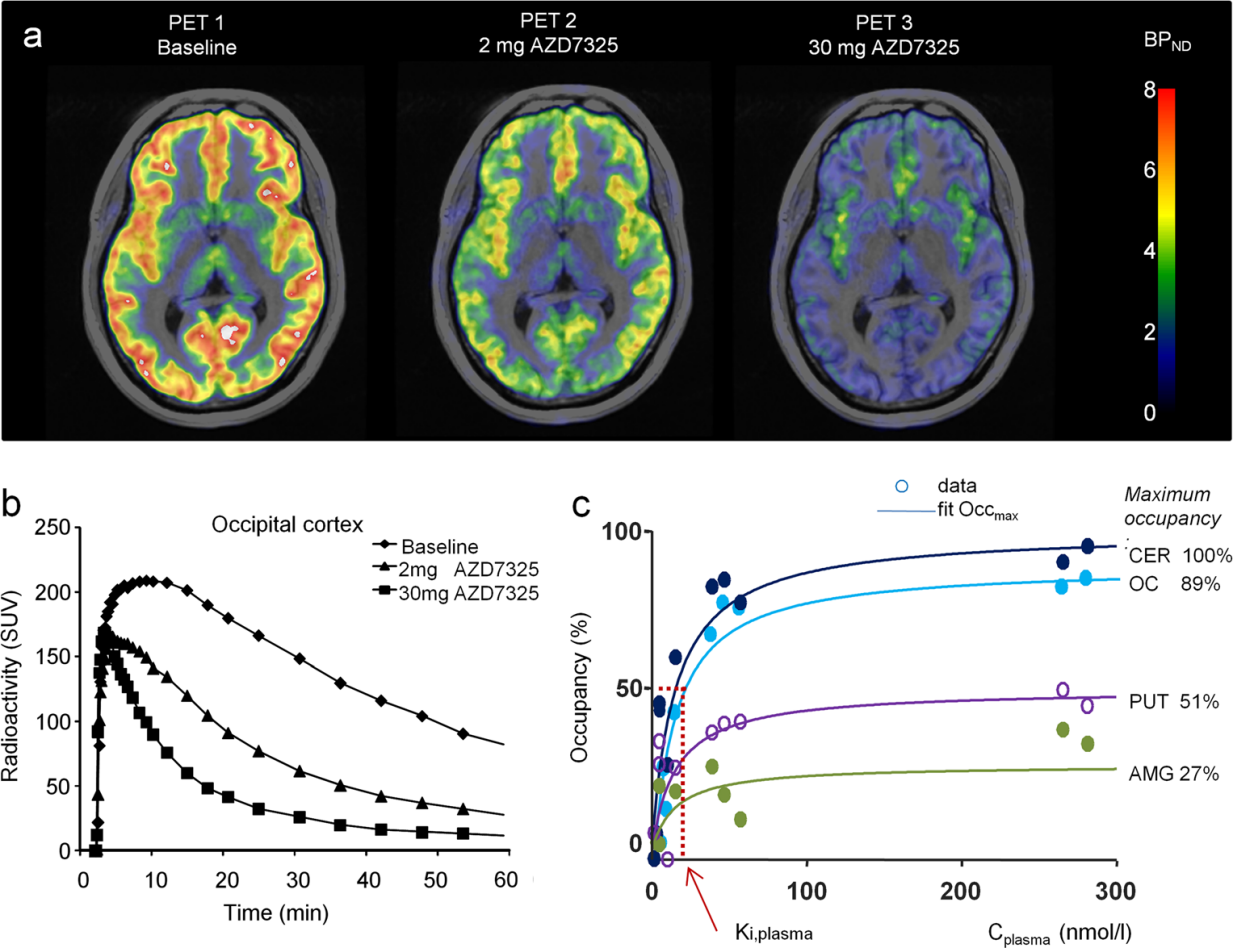
**a** Parametric PET images of [^11^C]flumazenil binding to brain GABA_A_ receptors at baseline and after oral administration of 2 and 30 mg of AZD7325 (PET images obtained using wavelet-aided parametric mapping (Cselényi et al. 2002) and fused with MR images; *BP*_*ND*_ binding potential; individual subject). **b** Time curves for radioactivity in the occipital cortex following administration of AZD7325 in the human subject. **c** Relationship between receptor occupancy and plasma exposure. Result of a model fit to the data (Eq. 2). The figure demonstrates regional maximum occupancy differences in relation to the differences in the fraction of GABA_A_ receptor subunits in the region (*CER* cerebellum, *OC* occipital cortex, *PUT* putamen, *AMG* amygdala). *K*_i,plasma_, best fit estimate, and 95% CI, obtained on the logarithmic scale, was 15 nmol/ml; 95% CI 10–24 nmol/l

**Fig. 3:**
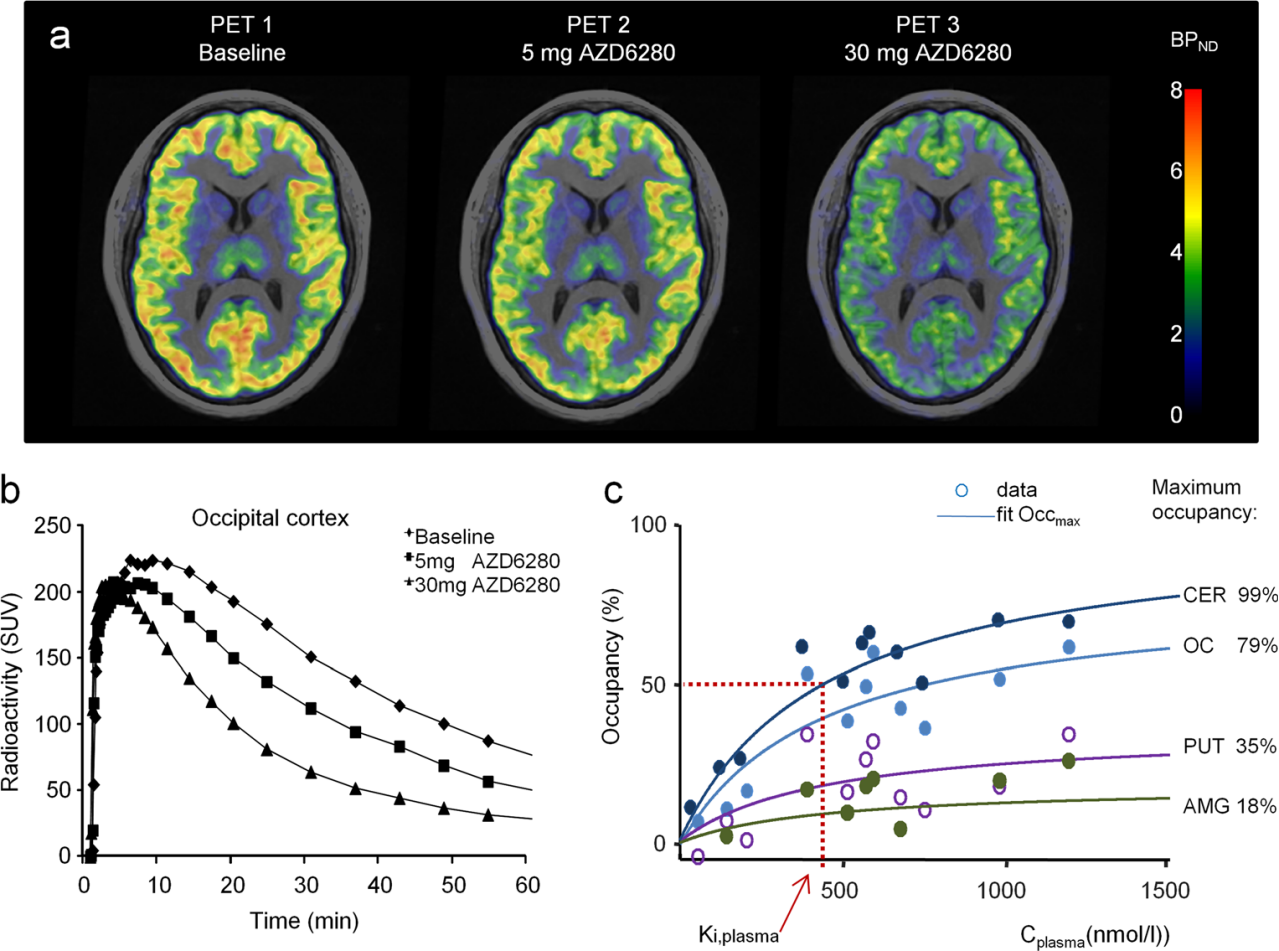
**a** Parametric PET images of [^11^C]flumazenil binding to brain GABA_A_ receptors at baseline and after oral administration of 5 and 30 mg of AZD6280 (PET images fused with MR images; *BP*_*ND*_ binding potential; individual subject). **b** Time curves for radioactivity in the occipital cortex after intravenous injection of [^11^C]flumazenil at baseline and following administration of AZD6280 in the human subject. **c** Relationship between receptor occupancy and plasma exposure. Result of a model fit to the data (Eq. 2). The figure demonstrates regional maximum occupancy differences in relation to the differences in the fraction of receptor subunits in the region (*CER* cerebellum, *OC* occipital cortex, *PUT* putamen, *AMG* amygdala). *K*_i,plasma_, best fit estimate, and 95% CI, obtained on the logarithmic scale, was 440 nmol/ml; 95% CI 197–982 nmol/ml

At baseline, specific binding of the non-selective radioligand [^11^C]flumazenil was the highest in the occipital cortex, followed by other cortical and subcortical regions, and lowest in the striatum (Tables 1 and 2; Figs. 2a and 3a). A dose-dependent decrease in [^11^C]flumazenil binding in all regions was observed after administration of AZD7325 and AZD6280 (Figs. 2b and 3b). The calculated GABA_A_ receptor occupancy at the highest doses of 20–30 mg AZD7325 reached over 80% in the occipital cortex and cerebellum. Correspondingly, at the maximum tolerated dose of 40 mg of AZD6280, receptor occupancy in the cortex and cerebellum was over 60%. For both compounds, maximal occupancy in putamen and amygdala was considerably lower than in occipital cortex and cerebellum (Tables 1 and 2; Figs. 2c and 3c).

### Estimation of *K*_i,plasma_

The relationship between GABA_A_ receptor occupancy and AZD7325 and AZD6280 plasma concentrations measured at time of PET data acquisition could be described by the hyperbolic function (Eq. 2). The AZD7325 plasma concentration required for 50% receptor occupancy (*K*_i,plasma_) for AZD7325 was 15 nmol/l and for AZD6280 440 nmol/l (Figs. 2c and 3c).

## Discussion

The present PET studies confirm GABA_A_ receptor occupancy in humans by the subunit-selective partial modulators AZD7325 and AZD6280. A high-resolution PET imaging system was used to examine receptor occupancy in the human brain in vivo. After administration of each compound, a marked reduction of [^11^C]flumazenil binding to the GABA_A_ receptors was observed, consistent with competitive binding at the benzodiazepine binding site.

The GABA_A_ receptor occupancy by AZD7325 and AZD6280 was dose dependent and could be described by a hyperbolic function indicating saturability. The plasma concentration corresponding to 50% of maximal GABA_A_ receptor occupancy (*K*_i,plasma_) was estimated and used as an index of affinity in vivo. The estimates of the *K*_i,plasma_ value of AZD7325 and AZD6280 were 15 and 440 nmol/l, respectively. Both compounds bind to human plasma proteins, leaving approximately 10% of the total plasma drug unbound. Neither compound is a P-gp substrate, and both compounds demonstrate good brain exposure in animals; thus, the 30-fold difference in affinity in vivo probably relates to differences in in vitro affinity (Suppl Table 1).

An important observation in the present PET studies was that high receptor occupancy, i.e., >70% for AZD7325 and >60% for AZD6280, could be reached by both compounds without obvious sedative effects. This characteristic of low or no hypnotic effect at high receptor occupancy was predicted by the translational studies in rodents for both compounds (Christian et al. 2015). A special focus was given to the examination of sedative effects across phase I clinical studies. In the single and multiple ascending dose studies (*ClinicalTrials.gov*, NCT00681317 and NCT00681915), sedation and cognition/information processing speed were assessed by patient-based monitoring using visual analog scale (VAS) alertness-sedation subscale, Modified Wilson Sedation Scale, and by Digit Symbol Substitution Test (DSST), and no significant relationship between AZD7325 or AZD6280 doses and alertness or cognitive performance was reported. There were just numerically higher sedation scores in VAS alertness scale compared to placebo at 1 h post-dose of AZD6280 and lower mean correct answers on DSST at 2 h after administration at dose ≥20 mg, i.e., at the exposure corresponding to receptor occupancy of >60% (AZ data on file). Moreover, no cognitive or hypnotic side effects were either observed in subjects examined in detail using neurophysiological test battery at the doses up to 80% RO for AZD7325 and up to 60% RO for AZD6280 (Chen et al. 2014, 2015). In contrast, at anxiolytic doses of non-selective positive allosteric modulators acting at the BZ site (diazepam, clonazepam, alprazolam, midazolam, lorazepam), significant drowsiness has been associated with low receptor occupancy, ranging from 2 to 30% (Shinotoh et al. 1989; Pauli et al. 1991; Sybirska et al. 1993; Malizia et al. 1996; Lingford-Hudges et al. 2005). More recently, for the partial GABA_A_ receptor agonists MRK-409 and TPA023, sedation has been reported at a broad range of receptor occupancy, from severe sedation at <10% occupancy (MRK-409) or lack of overt sedation up to 50% receptor occupancy (TPA023) (Atack et al. 2010, 2011a,b). One possible explanation for this broad range of tolerated levels of GABA_A_ receptor occupancy of novel partial GABA_A_ receptor agonists may be their intrinsic activity as suggested in the early studies comparing GABA_A_ receptor modulators (Bottlaender et al. 1994).

Other side effects, such as transient dizziness, euphoric mood, and hypoesthesia, were reported in clinical development of both studied compounds. AZD6280 and AZD7325 were screened in vitro in a broad panel of receptor binding sites to identify potential secondary targets. Specific binding and functional activity at melatonin type 1 and type 2 receptors (MT1, MT2) were found, more so for AZD6280 (Suppl Table 3). Sleep-promoting effects could, thus, be predicted, however were not observed in clinical studies. Hence, all the side effects reported could not be explained by secondary binding to other receptor and have been interpreted as benzodiazepine-like side effects. Importantly, they occurred at the exposure also corresponding to the high receptor occupancy for both compounds (>70% for AZD7325, >60% for AZD6280; adverse events in phase I clinical studies, Suppl Table 2).

The role of receptor occupancy in discriminating efficacy and side effects is best understood for antipsychotic drugs. PET studies using [^11^C]raclopride (Farde et al. 1986, 1992; Nyberg et al. 1995) have provided a large set of data showing that striatal D2 receptor occupancy at therapeutic dosing of typical antipsychotics is in the range of 70–80%, with increasing risk of extrapyramidal side effects at receptor occupancy over 80%. This concept has been successfully applied in the development of new generation sedation-free antihistaminergic compounds, by learning that histamine H1 receptor antagonists induce severe sedation at high occupancy (50–90%), whereas occupancy in the range of 5–30% is sufficient for therapeutic effects (Yanai and Tashiro 2007). The relationship between target receptor occupancy needed for the therapeutic efficacy for functional ion channel modulators is, however, far less well understood, and occupancy thresholds that would differentiate efficacy and side effects remain to be identified. Currently, the data set on clinical response or lack of it across novel selective BZs is too small to allow for more conclusive interpretation of relationship between the range of GABA_A_ receptor occupancy measured using [^11^C]flumazenil, intrinsic activity of compounds, and efficacy/side effect profile.

Although we have shown that high receptor occupancy can be achieved in humans without overt sedative affects or cognitive impairment, the question remains as to whether the intrinsic modulatory activity of these compounds is sufficient to produce a therapeutic effect. AZD7325, a compound with low intrinsic activity and highest tolerated receptor occupancy observed among BZs, showed only a weak anxiolytic effect in patients with generalized anxiety disorder (Chen et al. 2014, *ClinTrials.gov*). It is tempting to speculate that the reduced intrinsic activity of AZD7325 was sufficient to diminish sedative side effects but was too low to achieve optimal anxiolytic activity. Importantly though, clear pharmacodynamic effects of both compounds were observed in the human volunteer studies using psychometric tests and EEG (Chen et al. 2014, 2015). These effects were qualitatively and quantitatively distinct from those of lorazepam, and it is quite possible that the therapeutic effect of GABA_A_ receptor a2/3 subunit selective compounds may be more appropriate for other neuropsychiatric indications.

Recent efforts in pharmaceutical development of anxiolytics have focused on the development of subtype-selective GABA_A_ receptor modulators with reduced intrinsic efficacy. The combination of both changes may be beneficial but limits understanding of the role of each factor in isolation. Thus, the relative contributions of partial modulatory efficacy and subunit selectivity remain to be determined (Skolnick 2012; Sieghart 2015).

### Comments

For the quantitative analysis of [^11^C]flumazenil binding, the simplified reference tissue model (SRTM) was used with pons as reference region. GABA_A_ receptors in human brain are widespread, with no brain region that is devoid of receptors that could be used as reference. It has been shown, however, that GABA_A_ receptor density in pons is very low, appr. 5% of receptor binding in cortical regions (*B*_max_ in pons 2 pmol/g, in cortex 60–70 pmol/g, in subcortical regions 20–35 pmol/g, Hall et al. 1992). Though some displacement of radioligand in pons at high doses of agonists may occur, it has been used as a reference region, acknowledging potential underestimation of receptor occupancy (Abadie et al. 1996). Recent studies suggest that specific binding of [^11^C]flumazenil in the pons is negligible so that this region can be used as a reference region to obtain accurate BP_ND_ values (Odano et al. 2009).

Regional differences in the maximum GABA_A_ receptor occupancy were observed after administration of AZD7325 and AZD6280. The pattern of regional receptor occupancy may be explained by (i) the difference in the distribution of GABA_A_ receptor subtypes in the brain, (ii) the use of a non-selective radioligand, and (iii) the drug selectivity towards the GABA_A_ receptor subtypes. The data on the GABA_A_ receptor subunit distribution in the human brain are scarce, but some regional specificity has been suggested, e.g., GABA_A_ receptors containing α1 subunit are expressed predominantly in the cerebellum and thalamus, α5 appears mainly in the hippocampus combined with α2 and α3, and all four subunits are expressed in the cortex (Smith 2001; Fatemi et al. 2013; Waldvogel and Faull 2015). Imaging studies with the radioligand [^11^C]Ro15 4513, which has high affinity to α5 receptor subtype, have demonstrated that it is highly bound in the hippocampus, with a gradient of binding that decreases from the frontal cortex to the occipital cortex (Lingford-Hughes et al. 2002). Flumazenil is a benzodiazepine antagonist that has high affinity for the α1, α2, α3, and α5 receptor subtypes (*K*_i_ ∼1 nmol/l) and low affinity for the α4 and α6 receptor subtypes (*K*_i_ ∼150 nmol/l) (Sieghart 1995). AZD7325 and AZD6280 have a high affinity for the α1, α2, and α3 receptor subunits (in the range of 0.3 to 30 nmol/l) but low affinity to α5 subunit. Thus, it is tempting to relate the pattern of regional receptor occupancy observed in the present studies to the low affinity of AZD7325 and AZD6280 for the α5 subunit. It is important to note, though, that this interpretation has to be taken with caution, as the distribution of GABA_A_ receptor subunit combinations is still not fully revealed.

## Conclusions

The present PET studies confirmed that two novel α2/α3 receptor subtype-selective partial GABA_A_ receptor modulators bind in a saturable fashion to GABA_A_ receptors in the human brain. High GABA_A_ receptor occupancy by AZD7325 and AZD6280 could be reached at doses known to produce clear pharmacodynamic effects without clear sedation or cognitive impairment.

## Electronic supplementary material

Supplementary Table 1 (DOCX 23 kb).

Supplementary Table 2 (DOCX 17 kb).

Supplementary Table 3 (DOCX 13 kb).

Supplementary Figure 1 Structure of GABA_A_ modulators AZD6280 and AZD7325 (GIF 19 kb).

High resolution image (TIFF 763 kb).

## References

[CR1] Abadie P, Rioux P, Scatton B, Zarifian E, Barré L, Patat A (1996). Central benzodiazepine receptor occupancy by zolpidem in the human brain as assessed by positron emission tomography. Eur J Pharmacol.

[CR2] Alhambra C, Becker C, Blake T, Chang AH, Damewood JR, Daniels T (2011). Development and SAR of functionally selective allosteric modulators of GABAA receptors. Bioorg Med Chem.

[CR3] Atack JR, Wong DF, Fryer TD, Ryan C, Sanabria S, Zhou Y (2010). Benzodiazepine binding site occupancy by the novel GABAA receptor subtype-selective drug 7-(1,1-dimethylethyl)-6-(2-ethyl-2H-1,2,4-triazol-3-ylmethoxy)-3-(2-fluorophenyl)-1,2,4-triazolo[4,3-b]pyridazine (TPA023) in rats, primates, and humans. J Pharmacol Exp Ther.

[CR4] Atack JR, Hallett DJ, Tye S, Wafford KA, Ryan C, Sanabria-Bohórquez SM (2011). Preclinical and clinical pharmacology of TPA023B, a GABAA receptor α2/β3 subtype-selective partial agonist. J Psychopharmacol.

[CR5] Atack JR, Wafford KA, Street LJ, Dawson GR, Tye S, Van Laere K (2011). MRK-409 (MK-0343), a GABAA receptor subtype-selective partial agonist, is a non-sedating anxiolytic in preclinical species but causes sedation in humans. J Psychopharmacol.

[CR6] Baldwin DS, Anderson IM, Nutt DJ, Allgulander C, Bandelow B, den Boer JA (2014). Evidence-based pharmacological treatment of anxiety disorders, post-traumatic stress disorder, and obsessive–compulsive disorder: a revision of the 2005 guidelines from the British Association for Psychopharmacology. J Psychopharmacol.

[CR7] Bottlaender M, Brouillet E, Varastet M, Le Breton C, Schmid L, Fuseau C (1994). In vivo high intrinsic efficacy of triazolam: a positron emission tomography study in nonhuman primates. J Neurochem.

[CR8] Brown EG, Wood L, Wood S (1999). The medical dictionary for regulatory activities (MedDRA). Drug Saf.

[CR9] Chen X, Jacobs G, de Kam M, Jaeger J, Lappalainen J, Maruff P (2014). The central nervous system effects of the partial GABA-Aa2,3-selective receptor modulator AZD7325 in comparison with lorazepam in healthy males. Br J Clin Pharmacol.

[CR10] Chen X, Jacobs G, de Kam ML, Jaeger J, Lappalainen J, Maruff P (2015). AZD6280, a novel partial g-aminobutyric acid A receptor modulator demonstrates a pharmacodynamically selective effect profile in healthy male volunteers. J Clin Psychopharmacol.

[CR11] Christian EP, Snyder DH, Song W, Gurley DA, Smolka J, Maier DL (2015). EEG-β/γ spectral power elevation in rat: a translatable biomarker elicited by GABA(Aα2/3)-positive allosteric modulators at nonsedating anxiolytic doses. J Neurophysiol.

[CR12] Cselényi Z, Olsson H, Farde L, Gulyás B (2002). Wavelet-aided parametric mapping of cerebral dopamine D2 receptors using the high affinity PET radioligand [11C]FLB 457. NeuroImage.

[CR13] Farb DH, Ratner MH (2014). Targeting the modulation of neural circuitry for the treatment of anxiety disorders. Pharmacol Rev.

[CR14] Farde L, Nordström AL, Wiesel FA, Pauli S, Halldin C, Sedvall G (1992). Positron emission tomographic analysis of central D1 and D2 dopamine receptor occupancy in patients treated with classical neuroleptics and clozapine. Relation to extrapyramidal side effects. Arch Gen Psychiatry.

[CR15] Farde L, Hall H, Ehrin E, Sedvall G (1986). Quantitative analysis of D2 dopamine receptor binding in the living human brain by PET. Science.

[CR16] Fatemi SH, Folsom TD, Rooney RJ, Thuras PD (2013). Expression of GABAA α2-, β1- and ε-receptors are altered significantly in the lateral cerebellum of subjects with schizophrenia, major depression and bipolar disorder. Transl Psychiatry.

[CR17] Haefely W, Martin JR, Schoch P (1990). Novel anxiolytics that act as partial agonists at benzodiazepine receptors. Trends Pharmacol Sci.

[CR18] Hall H, Litton JE, Halldin C, Kopp J, Sedvall G (1992). Studies on the binding of [3H]flumazenil and [3H]sarmazenil in post-mortem human brain. Hum Psychopharmacol.

[CR19] Lammertsma AA, Hume SP (1996). Simplified reference tissue model for PET receptor studies. NeuroImage.

[CR20] Lingford-Hughes A, Hume SP, Feeney A, Hirani E, Osman S, Cunningham VJ (2002). Imaging the GABA-benzodiazepine receptor subtype containing the alpha5-subunit in vivo with [11C]Ro15 4513 positron emission tomography. J Cereb Blood Flow Metab.

[CR21] Lingford-Hughes A, Wilson SJ, Feeney A, Grasby PG, Nutt DJ (2005). A proof-of-concept study using [11C]flumazenil PET to demonstrate that pagoclone is a partial agonist. Psychopharmacology.

[CR22] Malizia AL, Gunn RN, Wilson SJ, Waters SH, Bloomfield PM, Cunningham VJ (1996). Benzodiazepine site pharmacokinetic/pharmacodynamic quantification in man: direct measurement of drug occupancy and effects on the human brain in vivo. Neuropharmacology.

[CR23] Malizia AL, Cunningham VJ, Bell CJ, Liddle PF, Jones T, Nutt DJ (1998). Decreased brain GABA(A)-benzodiazepine receptor binding in panic disorder: preliminary results from a quantitative PET study. Arch Gen Psychiatry.

[CR24] Nemeroff CB (2003). The role of GABA in the pathophysiology and treatment of anxiety disorders. Psychopharmacol Bull.

[CR25] Nyberg S, Nordström AL, Halldin C, Farde L (1995). Positron emission tomography studies on D2 dopamine receptor occupancy and plasma antipsychotic drug levels in man. Int Clin Psychopharmacol Suppl.

[CR26] Nutt DJ, Glue P, Lawson C, Wilson S (1990). Flumazenil provocation of panic attacks. Evidence for altered benzodiazepine receptor sensitivity in panic disorder. Arch Gen Psychiatry.

[CR27] Någren K, Halldin C (1998). Methylation of amide and thiol functions with [11C]methyl triflate, as exemplified by [11C]NMSP[11C]flumazenil and [11C]methionine. J Label Compd Radiopharm.

[CR28] Odano I, Halldin C, Karlsson P, Varrone A, Airaksinen AJ, Krasikova RN (2009). [18F]flumazenil binding to central benzodiazepine receptor studies by PET quantitative analysis and comparisons with [11C]flumazenil. NeuroImage.

[CR29] Olsen RW, Sieghart W (2009). GABAA receptors: subtypes provide diversity of function and pharmacology. Neuropharmacology.

[CR30] Pauli S, Farde L, Halldin C, Sedvall G (1991) Occupancy of the central benzodiazepine receptors during benzodiazepine treatment determined by PET. Eur J Neuropsychopharmacol 1–229

[CR31] Persson A, Ehrin E, Eriksson L, Farde L, Hedström CG, Litton JE (1985). Imaging of [11C]-labelled Ro 15-1788 binding to benzodiazepine receptors in the human brain by positron emission tomography. J Psychiatr Res.

[CR32] Pike VW, Halldin C, Crouzel C, Barré L, Nutt DJ, Osman S (1993). Radioligands for PET studies of central benzodiazepine receptors and PK (peripheral benzodiazepine) binding sites—current status. Nucl Med Biol.

[CR33] Puia G, Ducic I, Vicini S, Costa E (1992). Molecular mechanisms of the partial allosteric modulatory effects of bretazenil at gamma-aminobutyric acid type A receptor. Proc Natl Acad Sci U S A.

[CR34] Rudolph U, Crestani F, Benke D, Brünig I, Benson JA, Fritschy JM (1999). Benzodiazepine actions mediated by specific gamma-aminobutyric acid(A) receptor subtypes. Nature.

[CR35] Sternbach LH (1979). The benzodiazepine story. J Med Chem.

[CR36] Sybirska E, Seibyl JP, Bremner JD, Baldwin RM, al-Tikriti MS, Bradberry C (1993). [123I]iomazenil SPECT imaging demonstrates significant benzodiazepine receptor reserve in human and nonhuman primate brain. Neuropharmacology.

[CR37] Sieghart W (1995). Structure and pharmacology of gamma-aminobutyric acid A receptor subtypes. Pharmacol Rev.

[CR38] Sieghart W (2015). Allosteric modulation of GABAA receptors via multiple drug-binding sites. Adv Pharmacol.

[CR39] Shinotoh H, Iyo M, Yamada T, Inoue O, Suzuki K, Itoh T (1989). Detection of benzodiazepine receptor occupancy in the human brain by positron emission tomography. Psychopharmacol (Berl).

[CR40] Skolnick P (2012). Anxioselective anxiolytics: on a quest for the Holy Grail. Trends Pharmacol Sci.

[CR41] Smith TA (2001). Type A gamma-aminobutyric acid (GABAA) receptor subunits and benzodiazepine binding: significance to clinical syndromes and their treatment. Br J Biomed Sci.

[CR42] Tiihonen J, Kuikka J, Räsänen P, Lepola U, Koponen H, Liuska A (1997). Cerebral benzodiazepine receptor binding and distribution in generalized anxiety disorder: a fractal analysis. Mol Psychiatry.

[CR43] Trincavelli ML, Da Pozzo E, Daniele S, Martini C (2012). The GABAA-BZR complex as target for the development of anxiolytic drugs. Curr Top Med Chem.

[CR44] Varrone A, Sjöholm N, Eriksson L, Gulyás B, Halldin C, Farde L (2009). Advancement in PET quantification using 3D-OP-OSEM point spread function reconstruction with the HRRT. Eur J Nucl Med Mol Imaging.

[CR45] Waldvogel HJ, Faull RL (2015). The diversity of GABA(A) receptor subunit distribution in the normal and Huntington’s disease human brain. Adv Pharmacol.

[CR46] Yanai K, Tashiro M (2007). The physiological and pathophysiological roles of neuronal histamine: an insight from human positron emission tomography studies. Pharmacol Ther.

