# Supplementary material for: GABAA receptor occupancy by subtype selective GABAAα2,3 modulators: PET studies in humans
Source: Psychopharmacology (Berl). 2016 Dec 24;234(4):707–16. doi: 10.1007/s00213-016-4506-4 (PMC5263201; doi:10.1007/s00213-016-4506-4)
Supplement: Supplementary file 1 — (DOCX 23 kb). [file 213_2016_4506_MOESM1_ESM.docx]

**Supplemet Table 1.** Affinity *in vitro* (A), intrinsic activity (B) of GABA_A_ partial agonists and GABA_A_ receptor occupancy in humans

| 1. **Affinity** | | | | | |
| --- | --- | --- | --- | --- | --- |
| **Affinity in vitro at BZ binding site (Ki, nM)** | **α1** | **α2** | **α3** | **α5** | **Receptor occupancy/references, comments** |
| AZD6280 ^a^  (carboxamide) | 0.5±0.2 | 21±5 | 31±17 | 1680±650 | RO:60-70% (at 40mg, MTD)/ present study  Affinity: Christian et al 2015 |
| AZD7325 ^a^  (carboxamide) | 0.5±0.3 | 0.3±0.2 | 1.3±0.9 | 230±65 | RO:90-100% ( at ≥20mg, no MTD)/ present study  Affinity: Christian et al 2015 |
| MRK-409 ^b^ (triazolpyridazine) | 0.22±0.02 | 0.40±0.07 | 0.21±0.03 | 0.23±0.04 | <10 % (at 1mg, MTD)/ Atack et al 2010a |
| TPA-023B ^b^ (imidazotriazine) | 0.73±0.21 | 2.0±0.4 | 1.8±0.4 | 1.1±0.2 | 53% (at 1.5 mg, MTD 2mg)/ Atack et al 2010a |
| TPA-023 ^b^ (triazolpyridazine) | 0.27 | 0.31 | 0.19 | 0.41 | 35-65% (at 2mg, MTD) / Atack et al 2010a |
| Diazepam ^c, d, e^ (benzodiazepine) | (c ) 14  (d) 16.1±1.0 | 20  16.9±5.5 | 15  17.0±1.8 | 11  14.9 | RO:24%, at 30mg, sedation/ Pauli et al 1992  Affinity: c) Atack et al 1999, d) Sieghart 1995; |
|  | Cortex, cerebellum 16±4 to 21±1 , hippocampus 12.6±1.4 | | | | e) Dubinsky et al 2002 |
| Triazolam^d^ (benzodiazepine) | 1.8±0.4 | 1.2±0.2 | 3.0±0.7 | 1.2±0.3 | In NHP: 5-100% RO corresponds to 4-500 µg/kg/ Bottlaender et al 1994  Affinity: Sieghart 1995 |
| Zolpidem^c, d^ (imidazopyridine) | (c) 27  (d) 17±1.2 | 160  291±10 | 380  357±11 | >10 000  ND | RO: 21% (20mg, drowsiness), 15% at 10mg therapeutic dose /Abadie et al 1996  Affinity: c) Atack et al 1999, d) Sieghart 1995 |
| Alprazolam ^e^  (triazolbenzodiazepine) | Cortex, cerebellum 10.4 to 11.2, hippocampus 5 | | | | RO:16% (2mg/day, sedation) /Fujita et al 1999  Affinity: Dubinsky et al 2002 |
| Lorazepam ^e^  (benzodiazepine) | Cortex, cerebellum 1.8 ±0. 1 to 2.1 ± 0.1, hippocampus 8.6±4.6 | | | | RO: ≤10% (2 mg) / Lindfors-Hughes et al 2005, Atack et al 2010b  Affinity: Dubinsky et al 2002 |

*Notes:* Binding affinity is expressed as Ki – dissociation constant of an inhibitor. Different model system for the examination of the recombinant GABA_A_ receptors were used for the compounds presented in the table. For the drugs in use receptor occupancy cited is at therapeutic doses. RO- receptor occupancy

^a^ recombinant Sf9 membranes co-expressing GABA_A_ receptor subunits: α1; α1β2γ2, α2; α2β3γ2, α3; α3β3γ2, and α5;α5β3γ2. [^3^H]flunitrazepam. Data presented as mean (SD)

^b^ mouse fibroblast L(tk_) cells expressing human recombinant GABA_A_ receptors containing β3, γ2 plus either a1, a2, a3, a4, a5 or a6 subunits. [3H]flumazenil and [3H]Ro 15-4513. Data presented as mean (SEM)

^c^ human recombinant GABA_A_ receptors containing β3, γ2 plus either a1, a2, a3, a4, a5 or a6 subunits, radioligand [3H]Ro 15-1788

^d^ cited from Sieghart 1995, review

^e^ synaptosomal membranes from discrete regions of the rat brain using [3H]Flumazenil (cortex, cerebellum) or [3H]Ro15-4513 (hippocampus) (Dubinsky et al 2002)

| 1. **Intrinsic activity** | | | | | |
| --- | --- | --- | --- | --- | --- |
| **Relative efficacy (%)** | **α1** | **α2** | **α3** | **α5** | **Reference/comments** |
| AZD6280 (%DZ)* | 9±3 | 36±4 | 34±3 | 8±2 | Christian et al 2015 |
| AZD7325 (%DZ) | 0±1 | 19±1 | 17±1 | 11±1 | Christian et al 2015 |
| TPA-023  (%DZ)  (%CDP)** | 5±3  0 | 15±2  11 | 17±1  21 | 6±4  5 | Christian et al 2015  Atack et al 2010 /antagonist at α1 |
| MRK-409(%CDP) | 18 | 23 | 45 | 18 | Atack et al 2010/weak partial agonist at α1 |
| TPA023B(%CDP) | 3±1 | 38±4 | 50±2 | 37±4 | Atack et al 2010 |
| Diazepam° | 103±8 | 108±13 | 159±17 | 154±8.0 | Lippa et al 2005 |
| Triazolam *** | 132±6 | 255±11 | 270±13 | 165±8 | Sanna et al 2002 |
| Zolpidem *** | 100±5 | 198±20 | 217±34 | 15±5 | Sanna et al 2002 |

*Notes:** %DZ denotes for each compound at 1µM test concentration the positive modulatory effect on membrane current to a GABA EC10- concentration normalized as a percentage (mean ±SE) of that produced by a supramaximal concentration (1 µM) of diazepam. A voltage-clamp electrophysiological assay in *Xenopus* oocytes microinjected with specific combinations of GABA_A_ subunit cRNA (α1β2γ2, α2β3γ2, α3β3γ2, α5β3γ2) was used.

**%CPD denotes the maximum potentiation of the GABA EC20-induced currents measured using whole cell patch clamping in cell lines expressing human recombinant GABAA receptors containing β3γ2 plus either α1, α2, α3 or α5 subunits. Data are expressed as percentage (mean± SE) relative to the non-selective full agonist chlordiazepoxide

*** Data are means ±SE of EC50 values and the maximal percentage potentiation of GABA-evoked Cl _ currents in voltage-clamped *Xenopus*

oocytes expressing human α1β2γ2, α2β2γ2, α3β2γ2, α5β2γ2 GABAA receptors

**°** recombinant human GABAA receptors expressed in *Xenopus oocytes*.,cRNAs encoding GABAA receptor α1, 2-, 3-, or 5-, β 2, and γ2- or 3-subunits.GABA-gated inward currents measured using voltage clamp model. Maximum potentiantion of the GABA EC50-induced currents, Emax in %. (α1β2γ2, α2β2γ2, α3β2γ2, α5β2γ2)

*References limited to the Suppl Table 1:*

1.Lippa A, Czobor P, Stark J, Beer B, Kostakis E, Gravielle M *et al* (2005) Selective anxiolysis produced by ocinaplon, a GABA(A) receptor modulator. *Proc Natl Acad Sci U S A.* **102**: 7380-7385.

2.Atack JR, Smith AJ, Emms F, McKernan RM (1999) Regional differences in the inhibition of mouse in vivo [3H]Ro 15-1788 binding reflect selectivity for alpha 1 versus alpha 2 and alpha 3 subunit-containing GABAA receptors. *Neuropsychopharmacology* **20**: 255-262.

3.Atack JR, Wong DF, Fryer TD, Ryan C, Sanabria S, Zhou Y *et a*l (2010) Benzodiazepine binding site occupancy by the novel GABAA receptor subtype-selective drug 7-(1,1-dimethylethyl)-6-(2-ethyl-2H-1,2,4-triazol-3-ylmethoxy)-3-(2-fluorophenyl)-1,2,4-triazolo[4,3-b]pyridazine (TPA023) in rats, primates, and humans*. J Pharmacol Exp Ther* **332**: 17-25.

4.Dubinsky B, Vaidya AH, Rosenthal DI, Hochman C, Crooke JJ, DeLuca S *et al* (2002) 5-ethoxymethyl-7-fluoro-3-oxo-1,2,3,5-tetrahydrobenzo[4,5]imidazo[1,2a]pyridine-4-N-(2-fluorophenyl)carboxamide (RWJ-51204), a new nonbenzodiazepine anxiolytic. *J Pharmacol Exp Ther* **303**: 777-790.

5. Fujita M, Woods SW, Verhoeff NP, Abi-Dargham A, Baldwin RM, Zoghbi SS *et al* (1999) Changes of benzodiazepine receptors during chronic benzodiazepine administration in humans. *Eur J Pharmacol* **368**: 161-172.

6.Sanna E, Busonero F, Talani G, Carta M, Massa F, Peis M *et al* (2002) Comparison of the effects of zaleplon, zolpidem, and triazolam at various GABA(A) receptor subtypes. *Eur J Pharmacol* **451**:103-110.
