# Supplementary material for: GABAA receptor occupancy by subtype selective GABAAα2,3 modulators: PET studies in humans
Source: Psychopharmacology (Berl). 2016 Dec 24;234(4):707–16. doi: 10.1007/s00213-016-4506-4 (PMC5263201; doi:10.1007/s00213-016-4506-4)
Supplement: Supplementary file 2 — (DOCX 17 kb). [file 213_2016_4506_MOESM2_ESM.docx]

**Supplement Table 2.** Most common adverse events during treatment with AZD7325 and AZD6280

(summary from AstraZeneca Single Ascending Dose studies)

| **Adverse event** | **Number (%) of patients** | | | | | | | | | | | | | | | | | | | |
| --- | --- | --- | --- | --- | --- | --- | --- | --- | --- | --- | --- | --- | --- | --- | --- | --- | --- | --- | --- | --- |
|  | **Part A AZD6280** | | | | | | | | | **Part B AZD6280** | | | | | | | | | | |
|  | **Placebo** | | **1mg** | | **20mg** | | **40mg** | | | **Placebo** | | **5mg** | | | **30mg** | | | **60mg** | | |
| Number of subjects | 12 | | 8 | | 8 | | 8 | | | 11 | | 8 | | | 7 | | | 8 | | |
| Number of subjects with at least one adverse event | 6 (50) | | 2 (25) | | 6 (75) | | 6 (75) | | | 6 (55) | | 5 (64) | | | 2 (29) | | | 7 (88) | | |
| Most common adverse events (CNS related) |  | |  | |  | |  | | |  | |  | | |  | | |  | | |
| somnolence | 2 (17) | | 0 | | 1 (13) | | 3 (38) | | | 2 (18) | | 1 (13) | | | 2 (29) | | | 3 (38) | | |
| headache | 2 (17) | | 1 (13) | | 2 (25) | | 0 | | | 2 (18) | | 1 (13) | | | 0 | | | 1 (13) | | |
| dizziness | 0 | | 0 | | 0 | | 0 | | | 0 | | 2 (25) | | | 0 | | | 2 (25) | | |
| dreamy state | 0 | | 0 | | 0 | | 0 | | | 0 | | 0 | | | 0 | | | 1 (13) | | |
| abnormal coordination | 0 | | 0 | | 0 | | 0 | | | 0 | | 0 | | | 0 | | | 1 (13) | | |
| nystagmus | 0 | | 0 | | 0 | | 0 | | | 0 | | 0 | | | 0 | | | 1 (13) | | |
| euphoric mood | 0 | | 0 | | 0 | | 1 (13) | | | 0 | | 0 | | | 1 (14) | | | 0 | | |
| depersonalisation | 0 | | 0 | | 0 | | 0 | | | 0 | | 0 | | | 0 | | | 1 (13) | | |
| **Adverse event** | **Number (%) of patients** | | | | | | | | | | | | | | | | | | | |
|  | **Placebo** | **AZD7325** | | | | | | | | | | | | | | | | | | |
|  |  | **0.2 mg** | | **1mg** | | **5mg** | | **10 mg** | | **20 mg** | | **30 mg** | | **50 mg** | | | **75 mg** | | | **100 mg** |
| Number of subjects | 18 | 6 | | 6 | | 6 | | 6 | 6 | | 6 | | 6 | | | 6 | | | 6 | |
| Number of subjects with at least one adverse event | 5 (28) | 2 (33) | | 2 (33) | | 4 (67) | | 2 (33) | 1 (17) | | 3 (50) | | 1 (50) | | | 4 (67) | | | 4 (67) | |
| Most common adverse events (CNS related) |  |  | |  | |  | |  |  | |  | |  | | |  | | |  | |
| somnolence | 1 (6) | 0 | | 0 | | 1 (17) | | 0 | 0 | | 1 (17) | | 0 | | | 0 | | | 1 (17) | |
| dizziness | 0 | 0 | | 0 | | 0 | | 2 (33) | 0 | | 1 (17) | | 0 | | | 0 | | | 2 (33) | |
| hypoaesthesia | 0 | 0 | | 0 | | 1 (17) | | 0 | 0 | | 0 | | 0 | | | 0 | | | 0 | |
| paraesthesia | 0 | 0 | | 0 | | 0 | | 0 | 0 | | 0 | | 0 | | | 1 (17) | | | 0 | |
| euphoric mood | 1 (6) | 0 | | 0 | | 0 | | 0 | 1 (17) | | 1 (17) | | 0 | | | 3 (50) | | | 1 (17) | |
| anxiety | 0 | 0 | | 0 | | 0 | | 0 | 0 | | 0 | | 0 | | | 0 | | | 1 (17) | |

*Notes:* Adverse events (AEs) and medical/surgical history were classified according to the terminology of the Medical Dictionary for Regulatory Activities, MedDRA, version 11.0 (March, 2008) (Brown et al. 1999). Nervous system disorders and Psychiatric disorders pooled under CNS related AEs. Dose-escalation of AZD6280 was stopped at 60 mg dose.

AZD6280: cohort 1: 1, 20, 40 mg; cohort 2: 5, 30, 60 mg

AZD7325: cohort 1: 0.2, 1, 5, 10, 20, 30, 50, 75, 100 mg. Placebo given for 2 subjects at each dose level. No MTD reached.
