# Supplementary material for: GABAA receptor occupancy by subtype selective GABAAα2,3 modulators: PET studies in humans
Source: Psychopharmacology (Berl). 2016 Dec 24;234(4):707–16. doi: 10.1007/s00213-016-4506-4 (PMC5263201; doi:10.1007/s00213-016-4506-4)
Supplement: Supplementary file 3 — (DOCX 13 kb). [file 213_2016_4506_MOESM3_ESM.docx]

**Supplement Table 3.** Secondary receptor binding profile

| **Target** | **Species** | **IC50 (μM)** | |
| --- | --- | --- | --- |
|  |  | **AZD6280** | **AZD7325** |
| Melatonin MT1 | Human | 0.116 | 0.126 |
| Melatonin MT2 | Human | 0.664 | 1.37 |

*Note:* AZD6280 and AZD7325 were screened in a battery of approximately 160 different ligand binding assays at a concentration of 10 μM. Compounds were considered active against a specific target when they caused ≥50% inhibition in an assay with an IC50 <1µM. The only assays in which either compound was active were the melatonin Type 1 and Type 2 receptors. A functional effect was confirmed with human MT1 receptor GTPγS studies that demonstrated an EC50 value of 34 nM and Emax of 90% for AZD7325 and EC50 value of 19nM and Emax of 90% for AZD6280. The potential contribution of MT1 receptor activation to the effects of AZD7325 have not otherwise been investigated.

IC50- half maximal inhibitory concentration, EC50-drug concentration producing 50% of maximal effect, Emax-maximum possible effect, GTPγS binding assay - model for testing function al agonist activity of G-protein coupled receptors
