# Supplementary figures and images for: GABAA receptor occupancy by subtype selective GABAAα2,3 modulators: PET studies in humans
Source: Psychopharmacology (Berl). 2016 Dec 24;234(4):707–16. doi: 10.1007/s00213-016-4506-4 (PMC5263201; doi:10.1007/s00213-016-4506-4)

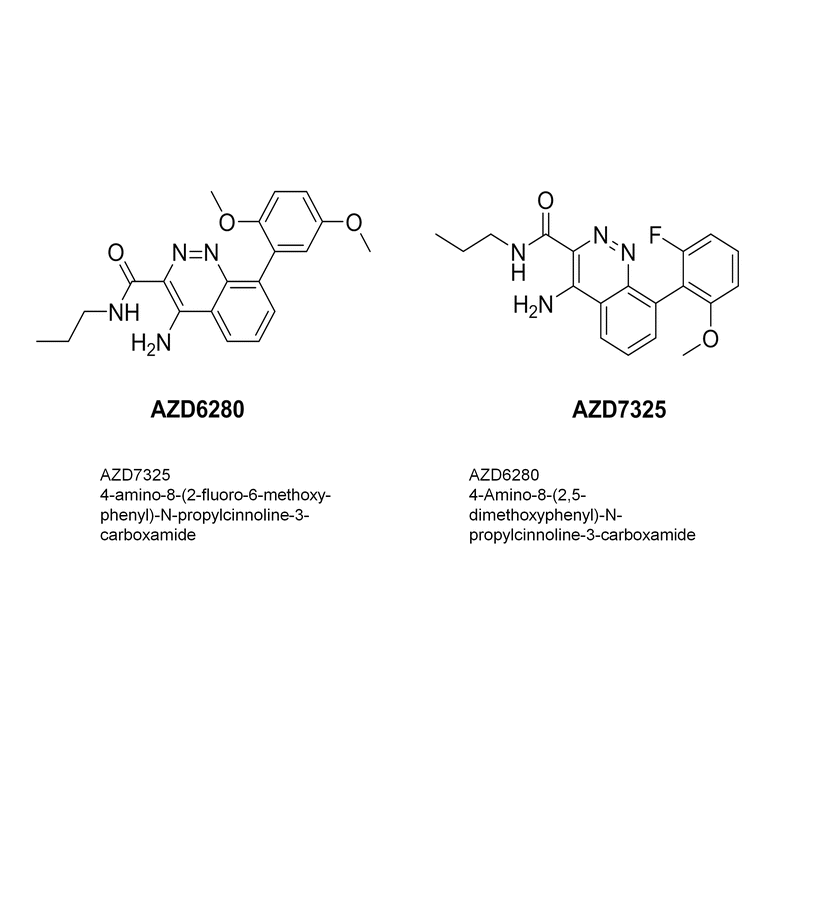

Supplement: Supplementary file 4 — Structure of GABAA modulators AZD6280 and AZD7325 (GIF 19 kb). [file 213_2016_4506_Fig4_ESM.gif]

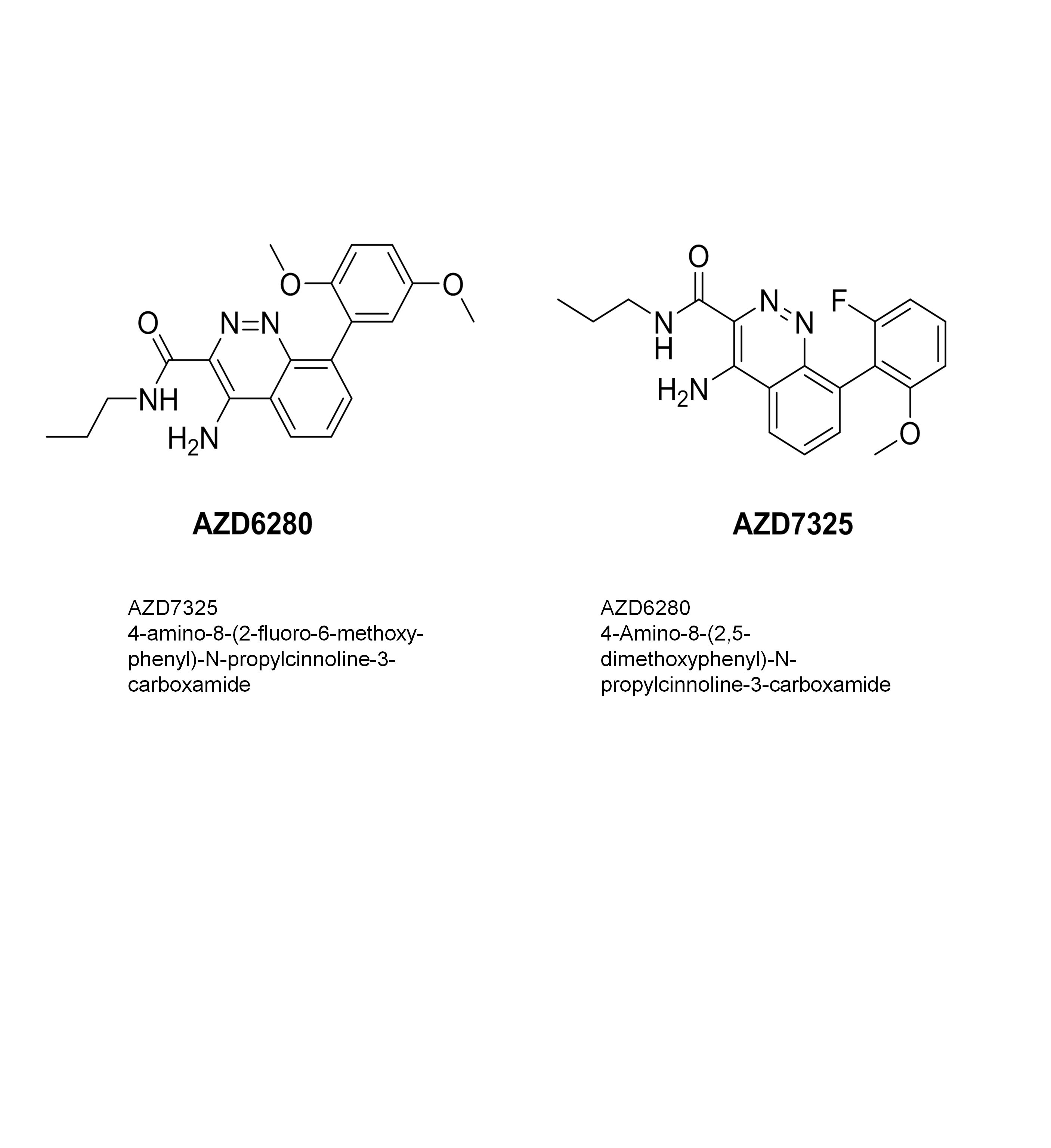

Supplement: Supplementary file 5 — High resolution image (TIFF 763 kb). [file 213_2016_4506_MOESM4_ESM.tif]
